# Managing Arsenic Pollution from Soil–Plant Systems: Insights into the Role of Biochar

**DOI:** 10.3390/plants14101553

**Published:** 2025-05-21

**Authors:** Qitao Su, Zhixuan Du, Xinyi Huang, Muhammad Umair Hassan, Faizah Amer Altihani

**Affiliations:** 1 Key Laboratory of Jiangxi Province for Biological Invasion and Biosecurity, School of Life Sciences, Jinggangshan University, Ji’an 343009, China; suqitao@jgsu.edu.cn (Q.S.); xinyihuang0049@163.com (X.H.); 2 Key Laboratory of Crop Physiology, Ecology and Genetic Breeding, Ministry of Education, Jiangxi Agricultural University, Nanchang 330045, China; 3 Department of Biology, College of Science, King Khalid University, Abha 61413, Saudi Arabia; ftehany@kku.edu.sa

**Keywords:** antioxidants, arsenic, biochar, health risks, microbes, remediation

## Abstract

Soil contamination with arsenic (As) is becoming a serious concern for living organisms. Arsenic is a nonessential metalloid for plants, humans, and other living organisms. Biochar (BC) is a very effective amendment to remediate polluted soils and it received great attention owing to its appreciable results. Arsenic toxicity negatively affects plant morph-physiological and biochemical functioning and upsurges the generation of reactive oxygen species (ROS), which negatively affect cellular structures. Arsenic toxicity also reduces seed germination and impedes plant growth by decreasing nutrient uptake, causing oxidative damage and disrupting the photosynthetic efficiency. Plants use different strategies like antioxidant defense and increased osmolyte synthesis to counteract As toxicity; nevertheless, this is not enough to counter the toxic impacts of As. Thus, applying BC has shown tremendous potential to counteract the As toxicity. Biochar application to As-polluted soils improves water uptake, maintains membrane stability and nutrient homeostasis, and increases osmolyte synthesis, gene expression, and antioxidant activities, leading to better plant performance. Additionally, BC modulates soil pH, increases nutrient availability, causes As immobilization, decreases its uptake and accumulation in plant tissues, and ensures safer production. The present review describes the sources, toxic impacts of As, and ways to lower As in the environment to decrease its toxic impacts on humans, the ecosystem, and the food chain. It concentrates on different mechanisms mediated by BC to alleviate As toxicity and remediate As-polluted soils and different research gaps that must be fulfilled in the future. Therefore, the current review will help to develop innovative strategies to minimize As uptake and accumulation and remediate As-polluted soils to reduce their impacts on humans and the environment.

## 1. Introduction

Heavy metal (HM) toxicity is serious for plants and humans because they negatively affect plant growth and cause health issues [1]. Arsenic (As) is a venomous metalloid for plants and it enters the food chain from crops grown on As-polluted soils [2,3,4]. Arsenic causes malfunctioning in the renal and cardiac system and immune systems, skin infections, and respiratory disorders [1,5,6]. It is widely found in the Earth’s crust, with an average concentration of 5 mg kg^−1^ [7,8]. Additionally, As is also found in 200 different mineral types, with 20% consisting of salts of sulfur and sulfides, 20 consisting of arsenides, arsenites, and silicates and the remaining 60% consisting of arsenates [9]. Among these, arsenite (As-III) has more poisonous and carcinogenic impacts than other forms, and it readily binds to plant tissues, causing a negative impact on plants [10]. Arsenic interferes with different metabolic processes, inhibits growth, and causes cell death [11,12,13]. Its accretion in plants upsurges ROS generation, which impairs photosynthesis efficiency and damages photosynthetic apparatus, antioxidant activities, cellular membranes, and deoxyribonucleic acid (DNA) [14,15]. It also alters plant metabolic functioning by interfering with carbon metabolism and disrupting nitrogen assimilation [16]. Further, As toxicity also reduces photosynthesis, stomatal conductance, carbon dioxide (CO_2_) intake, and respiration [17,18,19,20]. Plants use different mechanisms to counter the toxic impacts of As; for instance, they restrict the uptake of As by down-regulating the As transport genes, synthesizing As-chelating metabolites, increasing antioxidant activities and As sequestration into vacuoles [21,22,23,24].

Different methods like chemical precipitation, ion exchange, and phytoremediation are widely used to eliminate As from soil and water [8]. The former two techniques are very efficient in removing As; nevertheless, these techniques are costly and need energy and proper maintenance [25]. Conversely, phytoremediation is environmentally friendly, with broad applicability; however, it is a time-consuming technique [26,27]. Adsorption techniques like carbon-based materials, calcium-based biominerals, fly ash, biochar (BC), and graphene are also used for the removal of As and other toxic metals [28,29]. Biochar has emerged as an effective, environmentally friendly, and economical approach to remove HM toxicity, including As [28,29,30,31]. The application of BC has shown promising results for counteracting As toxicity and reducing its accretion in plants and soils [32]. Biochar has a higher surface area, water-holding capacity, and porosity, which makes it an excellent amendment to improve soil moisture, soil texture, and soil organic matter (SOM) and reduce the availability of toxic metals [33,34]. Biochar also increases microbial activities and soil fertility, thereby promoting plant growth by mitigating metal toxicity [35]. The modified BC has also shown tremendous ability to remediate As pollution in batch, soil, and pot studies [36,37,38]. This is linked with the fact that iron-modified BC effectively decreases the As bioavailability and its migration by causing the complexation, adsorption, and precipitation of As [39]. The aforementioned studies highlight the appreciable potential of BC to mitigate As toxicity. However, there is no comprehensive review available in the literature describing the mechanism mediated by BC to mitigate As toxicity. Thus, efforts are needed to investigate this mechanism to ensure safe and sustainable crop production. Thereby, the current review provides an in-depth understanding of different mechanisms mediated by BC to induce tolerance against As. Specifically, this review also explores the research gaps and future research directions for the remediation of As-polluted soils. This review provides new avenues and solutions to combat As pollution by using BC.

## 2. Arsenic, a Toxic Environmental Contaminant

**Figure 1:**
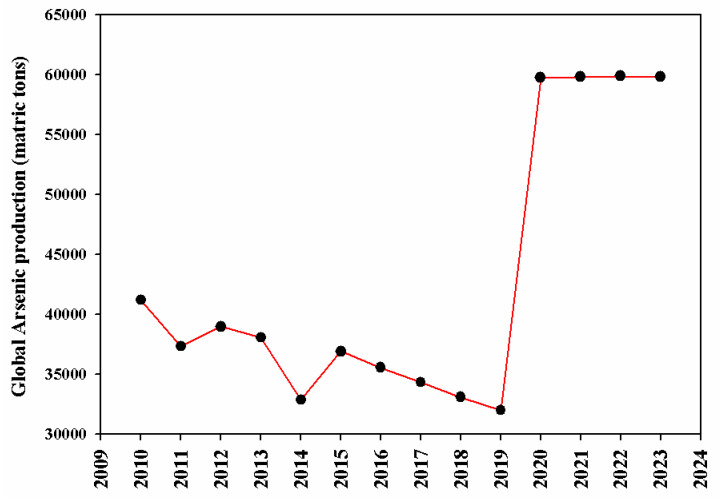
The global arsenic production from 2010 to 2023. The data used in the figure were collected from https://www.statista.com/statistics/797505/arsenic-worldwide-production/.

Arsenic is one of the most toxic pollutants among other metals; it is included in group-1 of human carcinogens recommended by the United States Environmental Protection Agency (US-EPA) and the International Agency for Research on Cancer (IARC). It is a serious threat to the ecosystem due to its serious toxic impacts and carcinogenic nature and abundant production (Figure 1) [40]. Arsenic is a nonessential metal for plants and living organisms and it causes serious changes in plant physiological, biochemical, molecular, and morphological responses even at very low concentrations.

## 3. Arsenic Occurrence and Distribution in the Environment

**Figure 2:**
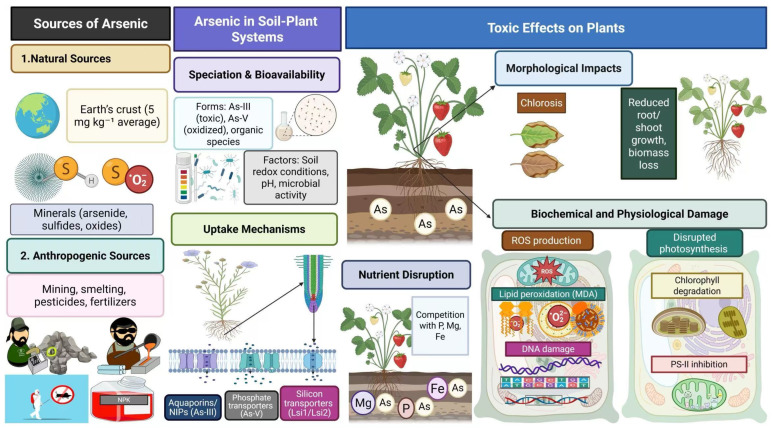
The sources of arsenic entry into soil, plants, and environment. Mining, smelting, pesticides, and fertilizers are important sources of As entry into environment. Arsenic toxicity decreases nutrient uptake, root growth, degrades chlorophyll, and damages photosynthetic apparatus, DNA, proteins, and lipids and increases ROS production, resulting in serious growth and yield losses.

Arsenic ranks as the 20th most abundant metal present in the Earth’s crust and it also holds 14th position in marine waters. It is a highly toxic metalloid for animals, humans, and plants and its concentration in the environment is continuously increasing (Figure 1). It exists in environments in different oxidation states such as As-III, As-0, and As-V. In our ecosystem, As is mostly present in the following forms: arsenite (As-III), arsenate (As-V), and mono-, di-, and tri-methyl arsenates [41]. It presents in soils from a few μg kg^−1^ to very high concentrations, such as 250,000 mg kg^−1^ [42]. The concentration of As in soils ranged from 5 to 10 mg kg^−1^ [43], while, in groundwater, the concentration of As ranged from 0.5 to 5000 μg L^−1^ [44]. However, WHO has set a threshold level of 10 μg L^−1^ in water (30). It is also a key component of >200 minerals and is found in different minerals such as arsenates, arsenites, arsenides and sulfides, and oxides. It is also found in association with different metals such as cobalt, gold, lead, mercury, nickel, and silver. The oxidation and reduction dissolution of As compounds sorbed onto pyrite minerals is a natural process of As distribution into the environment. Human activities annually release 52,000–1,120,000 tons As into the environment, which is a serious concern and needs dire attention [45]. Various human activities, including dyes, mining, pains, smelting, sludge, pharmaceuticals, chemical fertilizers (Figure 2), and pesticides, are the primary source of As entry into the environment and soil [43].

## 4. Arsenic Speciation and Bioavailability

Different factors including plant species, environmental conditions, and forms of As affect uptake and transportation in plants [46]. Generally, inorganic forms of As (As-III and As-V) are more dominant in soils than the organic forms [47]. The concentration of As-V can vary from 2.3 to 53 μM in uncontaminated to highly contaminated soil solutions [48]. This form of As also has a contribution of 73–96% in total As in aerobic soils [49]. Nevertheless, in reducing conditions, As-III is the predominant form and, in flooded soils, it has a share of 87–94% [39]. The concentration of As-III in flooded soil can vary from 0.01 to 3 μM, which is much higher than in non-flooded soils [50]. The conversion of As-V into As-III increases the mobility of As-III, thereby increasing As availability to plants [51]; however, it is significantly impacted by redox reactions and soil and water conditions [52,53].

## 5. Mechanisms of Arsenic Uptake and Transport Ion in Plants

Plants uptake As through both diverse pathways and transporters. Plants use high-affinity transporters involving the uptake of phosphate and they also facilitate As uptake. The structure of As-V is similar to phosphate, which enables As to enter plant roots. Phosphate transporters play a direct role in As uptake [43]; for instance, Cao et al. [54] noted the heterologous expression of *Pteris vittata* phosphate transporter (PvPht1; 3) in tobacco, which increased As translocation to shoots. This transporter has a strong affinity for absorption and translocation of As-V in tobacco plants. These authors also witnessed an up-regulation of GUS transporter gene in stele cells of tobacco, showing the potency of the aforementioned transporter in transport of As [54]. Moreover, Sun et al. [55] documented the over-expression of PvPht1, a gene that increases the translocation of As to tobacco shoots.

Aquaporins play an important role in the acquisition of As in plants and different families of aquaporin proteins are identified that mediate the uptake of As-III. Kamiya et al. [56] noted that nodulin-26-like intrinsic proteins (NIPs) are involved in the uptake of As-III. Further, Sun et al. [57] found that up-regulation of two NIP sub-family transporters, OsNIP1; 1 and OsNIP3; 3, considerably decreased As-III translocation and its accumulation in rice plants. Moreover, the expression of NIP protein (OsNIP3) significantly decreased As-III accumulation in roots and shoots of rice plants [58]. Two silicon transporters, namely Lsi1 and Lsi2, also play a role in the uptake and transport of As-III in plants. Lsi1 is a silicon transporter that plays a major role in As-III uptake pathway in rice. The up-regulation of Lsi-I in *Xenopus laevis* oocytes increased the acquisition of Asi-III, whilst Lsi-I in rice affects the influx of As-III inside the plant system [59].

## 6. Toxic Impacts of Arsenic on Plants

Arsenic negatively affects plant growth and development; however, the extent of toxicity depends on plant species, As types, and its accumulation in plants. It disturbs plant physiological, biochemical, and morphological processes, leading to poor growth [60]. Arsenic decreases root length, proliferation, cell divisions, and elongation (Table 1). Further, As-mediated reduction in root growth also decreases nutrient uptake, leading to a reduction in plant growth [61]. Arsenic (120 mg kg^−1^) induces chlorosis, cell death, and disturbs nutrient uptake, photosynthetic efficiency, growth, and biomass production [62]. It also decreases photosynthetic rate [63], photosynthetic efficiency, and endogenous nitric oxide (NO) levels [64,65]. Arsenic also increased malondialdehyde (MDA) and hydrogen peroxide (H_2_O_2_) production, which damage cellular membranes by increasing lipid peroxidation [66]. Moreover, it also decreases phosphorus uptake, leading to inhibited photosynthesis and cell death [67].

Arsenic decreases biomass and yield by decreasing leaf area and causing chlorosis, discoloration (Table 1), leaf senescence, and defoliation [68]. Talukdar et al. [69] tested the impacts of different concentrations of As (0, 10, 20, 30, and 40 mg L^−1^) on the performance of *Trigonella foenum-graecum* L. and *Lathyrus sativus* L. They witnessed a significant reduction in germination, growth, and biomass with increasing As concentration in the growing medium [69]. The induction of oxidative stress is one of the foremost impacts of As (50 µM As), which causes changes in plant functioning [70].

**Table 1:** Effect of arsenic stress on growth, physiological functioning, nutrient homeostasis, osmolyte synthesis, and antioxidant activities of different plant species.

Plant Species	As Concentrations	Growth Media	Major Effects	References
Maize	50 mg kg^−1^	Pot	Arsenic toxicity decreased RWC, chlorophyll and carotenoid synthesis, stomatal size, and density and increased As accumulation in maize shoots.	[71]
Maize	3.2 mg L^−1^	Pot	Arsenic decreases leaves, plant height, stem girth, pedunle length, chlorophyll and carotenoid synthesis, APX, and SOD activity and increased lipid peroxidation, phytochelatins production, and soil As availability.	[72]
Maize	120 mg kg^−1^	Pot	Arsenic toxicity decreased the plant morphological performance, phosphorous accumulation, chlorophyll synthesis, transpiration rate, stomatal conductance, and water use efficiency and increased MDA and ROS production and As accumulation.	[73]
*Date Palm*	1 mM	Pot	Arsenic stress decreased root and shoot growth and biomass production, and chlorophyll synthesis and increased oxidative damages, lipid peroxidation, MDA, and O^2•−^ production.	[74]
Mustard	2 mM	Pot	Arsenic stress decreased plant fresh weight (35–47%), root length (38%), shoot length (39%), and chlorophyll synthesis (9–16%) and increased thiobarbituric acid reactive substances (53–125%), H_2_O_2_ production, and nonprotein thiols.	[5]
Mustard	75 mg kg^−1^	Pot study	Arsenic toxicity decreased root (25%) and shoot (27%) dry weight, plant height (39%) and leaf area (23%), chlorophyll-a (12%), chlorophyll-b (15%), carotenoid (6%), SOD (65%), POD (23%), APX (28%), GR (32%), and GST (46%) and increased ROS and MDA production. As also increased non-protein thiols, cysteine and phytochelatins, and accumulation and translocation of As.	[75]
Wheat	2 mM	Petri dish	Arsenic toxicity reduced seed germination, seedling growth, chlorophyll synthesis, and antioxidant activities (APX, POD, SOD, and CAT) and increased the production of TBARS, lipid peroxidation, and H_2_O_2_.	[76]
Wheat	100 μM	Pot	Arsenic stress increased EL, antioxidant activities, MDA and H_2_O_2_ production, and accumulation of osmolytes. Further, As also decreased RWC, photosynthetic efficiency, chlorophyll synthesis, stomatal conductance, and transpiration rate.	[77]
Wheat	60 mg kg^−1^	Pot	Arsenic toxicity inhibited the plant growth, productivity, photosynthetic pigments, oxidative damages, and As accumulation in roots and shoots and increased APX, SOD, and POD activities.	[78]
Wheat	70 μM	Pot	Arsenic toxicity declined plant height, tillers, spike length, crop growth rate, stomatal conductance, and soil N, P, and K availability, and increased EL and As accumulation in wheat tissues.	[79]
Wheat	2.02 mg kg^−1^	Pot	Arsenic toxicity decreased plant height, plant biomass, spike length, grain weight, chlorophyll synthesis, and SPAD contents and increased MDA, EL, and H_2_O_2_ production, and As accumulation in roots, shoots, and grains.	[80]
Rice	70 µM	Pot	Arsenic stress decreased shoot (53%) and root length (64%) and their biomass (51–67%), photosynthetic rate (49%), stomatal conductance (2%), CO_2_ concentration (51%), MDA (33%) and transpiration rate (38%), tissue nitrogen (12%), potassium (16%), and zinc (18%) concentration and increased SOD (28%), POD (49%), and CAT (46%) activities.	[81]
Rice	2 mg L^−1^	Hydroponic	Arsenic decreased root and shoot growth and biomass, chlorophyll synthesis (27.3%), SOD activity (34.46%), increased EL (8.8–15.4%), and increased root and shoot As concentration.	[82]
Rice	10 μmol L^−1^	Hydroponic	Arsenic toxicity decreased root and shoot elongation, biomass production, root surface area, grain weight, and grain yield and increased As accumulation in plant tissues.	[83]
Rice	150 μM	Hydroponic	Arsenic toxicity declined root length (21%), shoot length (11%), fresh biomass (35%), dry biomass (36%), chlorophyll synthesis (55%), and anthocyanins (25%) and increased Mg concentration (61%), AAO activity (36%), and proline synthesis (97%).	[84]
Rice	1 mM	Pot	Arsenic toxicity decreased plant dry biomass (35%), RWC (27%), and chlorophyll synthesis (44%) and increased As accumulation, proline synthesis (177%), MDA (27%), H_2_O_2_ (89%) production, and increased antixidant activities.	[85]
Spinach	100 mg kg^−1^	Pot	The plant growth, chlorophyll synthesis, chlorophyll fluorescence, free amino acid synthesis, and tissue zinc and manganese synthesis were significantly decreased under As stress.	[86]
Tomato	3.2 mg L^−1^	Peat moss	Arsenic decreased shoot and root dry biomass by 8.53% and 11.57%, Ca concentration in leaves (43.7%) and fruits (38.31%), and increased As accumulation, H_2_O_2_ production, and flavonoids contents.	[87]
Barley	150 μm	Hydroponic	Arsenic treatment decreased shoot length (33.4%), root length (27.9%), shoot (36.3%) and root (25.6%) fresh biomass, chlorophyll synthesis, and fluorescence and increased MDA and ROS production, As accretion in roots and shoots, and decreased Ca uptake. Further, As toxicity also increased the expression of As transport genes.	[88]
Bamboo	250 μM	Tissue culture chamber	Arsenic accumulation, translocation factor, bioaccumulation factor, soluble sugars, and membrane stability were decreased under As stress. Further, As increased ROS production and antioxidant activities.	[89]
Lentil	100 mg kg^−1^	Pot	Arsenic toxicity decreased soil phosphorous, potassium, nitrogen and sulfur availability, root and shoot length, and biomass production and increased As uptake and accumulation in roots and shoots of lentil.	[90]

RWC: relative water contents, APX: ascorbate peroxidase, SOD: superoxide dismutase, As: arsenic, MDA: malondialdehyde, H_2_O_2_: hydrogen peroxide, POD: peroxidase, GR: glutathione reductase, GST: glutathione S-transferases, ROS: reactive oxygen species, EL: electrolyte leakage, N: nitrogen, P: phosphorus, K: potassium, CO_2_: carbon dioxide, AAO: ascorbate oxidase, Ca: calcium, Mg: magnesium.

Arsenic also triggers oxidative damage through the production of oxidative stress by repressing the antioxidant enzymes [91]. It increases ROS in mitochondria [92], which impedes succinic dehydrogenase activity [93]. Arsenic also triggers ROS production by nicotinamide adenine dinucleotide phosphate (NADPH) oxidase and it also disrupts nitric oxide synthase (NOS) activity and arginine metabolism, resulting in ROS production [93]. This causes oxidation of membranes, and damages lipids, leading to the generation of fatty acid radicals and MDA [94]. The oxidation of membranes by As also leads to leakage of cellular contents and electrolytes [95]. Arsenic toxicity also damages photosynthetic apparatus, activity of PS-II, and chlorophyll synthesis [96]. Magnesium is an important nutrient involved in chlorophyll synthesis and it may be replaced with an As atom in the tetrapyrrole ring, which impacts pathways involved in chlorophyll synthesis [16]. Arsenic also slows the Calvin cycle, which causes a substantial reduction in plant photosynthetic efficiency [96].

Arsenic induces toxicity in mitochondria in diverse ways; for instance, it enters the mitochondria by mitochondrial dicarboxylate transporters [97]. This, in turn, inhibits complex-1 of the mitochondrial electron transport chain and increases ROS, which damages proteins and causes lipid peroxidation [15]. Arsenic also competes with phosphate ions, leading to pyruvate production without net adenosine triphosphate (ATP) production. Plant growth, development, and stress resistance depend on lipid metabolism. The oxidation of lipids is a very destructive process mediated by As and As toxicity often leads to membrane damage and electrolyte leakage [98]. Excessive ROS production causes oxidation of lipid molecules in membranes [99]. Moreover, the oxidation of membranes also leads to the production of MDA and hydroxynonenal [100].

The findings of Yu et al. [101] showed that As-III (100 μM) toxicity modulated the expression of 59 genes linked with the production of lipids. Arsenic (25 μM AsV or AsIII) also affects lipid signaling through the induction of phosphatidic acid signal in addition to peroxidation of lipid peroxidation [102]. Phosphatidic acid is a crucial precursor involved in the manufacturing of triacyl-glycerols and glycerol-phopsholipids [103]. Inorganic As has a higher affinity for sulfhydryl groups in proteins, which causes membrane damage and cell death. Arsenic toxicity also decreases protein synthesis [87] by decreasing the activity of RNAase and protease, which leads to impaired hydrolysis of both RNA and proteins [104]. Additionally, As also oxidizes proteins leading to the formation of protein carbonyls [60].

## 7. Mechanisms Mediated by Biochar to Mitigate Arsenic Toxicity

Biochar is an alkaline material with abundant functional groups that directly stabilize toxic metals in soil by adsorption, complexation, and precipitation [105,106]. Biochar directly affects plant functioning, thereby mitigating the toxic impacts of heavy metals. It also indirectly stabilizes toxic metals in soil by modifying soil properties and composition–transformation processes. Biochar has shown promising results in mitigating As toxicity both in soil and plants. It mitigates As toxicity through indirect and direct mechanisms and details of both these mechanisms are given below.

### 7.1. Direct Mechanisms Mediated by Biochar to Mitigate as Toxicity

#### 7.1.1. Biochar Improves Water Uptake and Maintains Membrane Stability to Counter Arsenic Toxicity

**Figure 3:**
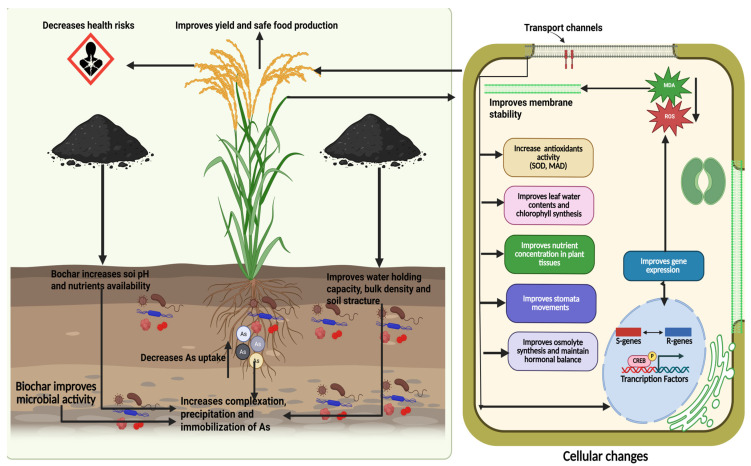
Biochar application improves plant growth and development by different mechanisms. The application of biochar to As-contaminated soils improves soil fertility, microbial growth, nutrient uptake, causes immobilization and complexation of As, decreases ROS production and improves stomatal conductance, membrane stability, osmolyte synthesis, hormonal balance, and gene expression and, thereby, improves plant growth.

Arsenic (As-V and As-III, 25 μM) decreases leaf water contents by decreasing water uptake due to inhibited root growth [11]. The application of BC increases leaf water contents in As-polluted soil (600 mg kg^−1^) by increasing nutrient uptake (Figure 3) due to better root growth [107]. Arsenic toxicity also damages the cellular membrane by increasing the production of free radicals, MDA, and H_2_O_2_ production. However, BC application improves membrane stability of membranes by decreasing MDA and H_2_O_2_ production through substantial increase in ascorbate peroxide (APX), catalase (CAT), and superoxide dismutase (SOD) [98]. Recently, Liao et al. [108] found that application of 0.5% BC to arsenic-polluted soil (600 mg kg^−1^) decreased electrolyte leakage by ~ 19% under 600 mg kg^−1^ As stress. The same BC application increased the relative water contents (RWC) by ~ 50% compared to the control [60]. Irshad et al. [109] witnessed that BC added to As- (600 mg kg^−1^) and Cd-polluted soils protected the membrane integrity by decreasing As and Cd accretion in the roots and leaves of rice [109]. Moreover, Majumdar et al. [37] found that rice-residue-based BC minimized As toxicity and led to higher cellulose production, resulting in better ultra-structure of xylem, phloem, and bundle sheath.

#### 7.1.2. Biochar Improves Synthesis of Potential Osmolytes and Increases Antioxidant Activity to Alleviate Arsenic Toxicity

Osmolyte accumulation plays a crucial role in mitigating adverse impacts of heavy metals [110]. Biochar application improves osmolyte accumulation, which helps to counter As toxicity. The findings of Hakeem et al. [107] showed that As toxicity (600 mg kg^−1^) decreased proline synthesis and soluble sugars. This decrease was associated with alterations in carbohydrate metabolism [111]. Nevertheless, BC application enhanced the soluble sugars, which was linked with starch degradation, which led to accretion of sugars and maintenance of better osmotic balance in Pb-polluted soil [107]. Alwutayd et al. [112] indicated that BC increased proline synthesis by increasing gene expression, which helped in counteracting the As (100 µM) toxicity.

Biochar application increases nitrate (NO^3−^) concentration and nitrate reductase (NR) activity. This increase in nitrate accumulation plays a crucial role in amino acid synthesis [113]. The increase in protein and amino acid synthesis is very effective in increasing stress in plants [114]. Biochar mitigated ROS excessive accumulation produced by As by converting ammonium (NH_4_^+^) into glutamate and glutamine through glutamate dehydrogenase (GDH) action rather than an alternative GS-GOGAT pathway. Cañas et al. [115] found that an increase in GDH synthesis increases the production of oxoglutarate, aspartate, proline, tryptophan, and lysine. Biochar application replenishes the TCA cycle and protects cell viability by over-activation of deaminating GD [116].

Antioxidant defense is an important strategy used by plants to counter the toxic impacts of As toxicity. Alam et al. [117] found that mung bean plants grown in As (30 mg kg^−1^) polluted soils showed an increase in CAT activities, which was further increased with BC. Shabbir et al. [118] also noted that BC application enhanced the antioxidant activities of quinoa plants grown in As- (30 mg kg^−1^, As-III) and salts-contaminated soil. Further, Wen et al. [119] also found that BC applied to contaminated soils increases the photosynthetic pigments and antioxidant activities, which countered the toxicity of As. Recently, it was found that 0.5% BC application decreased the POD, SOD, and CAT activities by 2%, 4%, and 4%, respectively, which was linked with reduced As accumulation [108]. Biochar stimulated defense pathways based on rates of BC application. The application of BC applied at higher rates decreased H_2_O_2_ and thiobarbituric-acid-reactive substance (TBARS) production by increasing the activities of CAT and SOD and As-polluted soils [107]. The application of BC could also promote the activities, APX, dehydroascorbate reductase (DHAR), monodehydroascorbate reductase (MDHAR), and ascorbic acid (AsA), which decreases ROS production and protects the plants from oxidative damage [116] (see Table 2).

Irshad et al. [109] documented that BC addition to As-polluted soil increased CAT and SOD activity and decreased CAT activity. BC application also improves the AsA-GSH activities, which maintains redox balance, thus protecting the plants from oxidative damage [112]. Ascorbic acid and glutathione (GSH) are strong buffer agents that regulate redox balance and support different processes in plants. The increase in activities of both ASA and GSH increases tolerance against As tolerance by maintaining redox balance and scavenging ROS [136].

#### 7.1.3. Biochar Ensures Better Photosynthetic Efficiency and Gene Expression Under Arsenic Toxicity

Arsenic toxicity decreases chlorophyll and carotenoid synthesis by interfering with N metabolism and increasing the activity of chlorophyllase [37]. Biochar application improves chlorophyll and carotenoid synthesis under As (Figure 3) by protecting the photosynthetic apparatus and increasing quantum yield [137]. Arsenic stress decreases photons captured by reaction centers in PS-II and it also decreases the efficiency of PS-II [116]. The application of BC increases photosynthetic efficiency, inter-cellular CO_2_, and transpiration, which favors plant growth under As stress [105]. Biochar maintains chlorophyll synthesis and improves gas exchange traits by decreasing As uptake [105]. Biochar improves iron (Fe) uptake, which plays an imperative role in chlorophyll synthesis, d electron transportation [138], and efficiency of PS-II [139,140]. Magnesium is an important component of chlorophyll structure [128], while potassium (K) also contributes to the formation of chlorophyll precursor and prevention of chlorophyll decomposition [141,142]. Biochar application under As-polluted soils (10 mg kg^−1^) increased nutrient uptake of iron (Fe) and magnesium (Mg: Table 2), leading to an increase in chlorophyll synthesis and photosynthetic efficiency. Besides this, BC also mitigates oxidative damage by decreasing ROS production, which protects the photosynthetic apparatus from As-induced oxidative damages [143].

Gene expression plays a critical role in stress tolerance. Biochar increases gene expression, which helps to counteract As toxicity. Recently, the effect of BC was studied on two rice cultivars (Shatabdi and IR-64) grown in flooding and alternate wetting and drying conditions (AWD). The application of BC under AWD increased the interaction beween As, silicon (Si), and phosphorous (P), with a concomitant down-regulation of Si (*OsLSi1* and *OsLSi2*) and P (*OsPT1* and *OsPT2*) uptake genes in Shatabdi and IR-64. They also found that BC application also promoted the synthesis of phytochelatins (PCs), which led to up-regulation of *OsABCC1* gene that ensured a better complexion of As and PCs. The formation of the aforementioned complexes reduces As uptake and its subsequent accumulation in plant tissues [144]. Xu et al. [145] exposed cotton plants to Cd, Pb, and As supplemented with BC (10 g kg^−1^). They found that BC maintained ionic homeostasis by increasing the expression of metal transport genes, including *ABC*, *HIPP*, *NRAMP3*, *PCR*, and *ZIP*, and genes linked with carbon metabolism and plant membranes. Biochar also down-regulates the genes associated with photosynthesis and maintains redox homeostasis by increasing CAT, POD, and SOD activities and expression of genes linked with these antioxidants [145].

### 7.2. Indirect Mechanisms Mediated by Biochar to Mitigate as Toxicity

#### 7.2.1. Biochar Modulates Soil pH and Improves Nutrient Availability to Counter Arsenic Toxicity

Soil pH plays a crucial role in As availability and BC increases soil pH and dissolved organic carbon (DOC), which decreases the availability of As to plants and in soil. However, the effect of BC on soil pH and DOC largely impacts the type of feedstock and properties of BC [146]. Recently, Kumar et al. [147] found that BC increased soil pH and decreased the bulk density of As-polluted soil (100 mg kg^−1^). Further, Wong et al. [148] noted that rice-residue-based BC (1%) increased the nutrient availability and improved the structure of root, hydraulic conductivity, and water retention. This was linked with increased organic carbon availability, which improved the soil structure and led to better growth in As-V-polluted soil. Biochar also improves K uptake acquisition, which increases ribulose-1,5-bisphosphate carboxylase/oxygenase (RuBiSCo) activity, gas exchange properties, and activates different enzymes linked with energy metabolism under As-polluted soils [149]. Calcium (Ca) regulates stomatal movement and electron transport, while Mg and Fe play a crucial role in chlorophyll synthesis [19]. Biochar application significantly increases the photosynthetic rate and subsequent growth in As-V-contaminated soils by increasing the uptake of Ca, Mg, and Fe [120].

Hafeez et al. [119] showed that BC and iron-modified BC increased the soil’s total organic carbon [119]. Kumar et al. [150] prepared BC from different biomasses like wheat, rice straw, kitchen waste, leaf litter, *Lantana camara*, orange peel, and walnut and applied it to As-polluted soil (100 mg kg^−1^). They applied all the BC at different rates, such as 0, 2.5, 5, and 7.5%, and found a dose-dependent impact of BC on soil properties and As accumulation. Biochar used at a rate of 7.5% enhanced CEC (62%), soil porosity (32%), water holding capacity (86%), total carbon (2.5%), total nitrogen (11%), and total phosphorus (3 times) and decreased mobile As (38%), leachable As (53%), and available As by 56%. Further, the same BC application enhanced the biomass production in gram and coriander plants by 61% and 177% and decreased As accumulation by 56% and 55%, respectively, in aerial plant parts [142]. Moreover, Dias et al. [151] found that BC increased K, manganese (Mn), phosphorus (P), and sulfur availability in mining tailings of As, leading to a substantial decrease in As availability owing to competition between As and nutrient uptake.

#### 7.2.2. Biochar Causes Arsenic Immobilization and Decreases Its Uptake to Counter Arsenic Toxicity

Biochar application reduces As uptake and causes its immobilization. For instance, Hakeem et al. [107] showed that BC applied to As-treated soil (200 mg kg^−1^) increased root and shoot translocation index (TI) by 124.4% and 83.69%, respectively. These authors also noted that root As concentration was increased to 61.95 and 93.80 mg g^−1^, while shoot As concentration was increased to 16.98 and 40.03 mg g^−1^ at 200 and 600 mg kg^−1^ As stress. Nevertheless, BC decreased the root concentration to 26.91 and 46.2 mg g^−1^ DM at both levels of As, while it decreased shoot 8.82 and 16.84 mg g^−1^ [105]. Xu et al. [152] found that BC application reduced As uptake by roots and shoots of soybeans. This reduction in As (60 mg L^−1^, As-III) uptake with BC was linked to the strong adsorption capacity of BC that enhances hydrodynamic size and induces As precipitation [57].

Ibrahim et al. [76] found that application of cotton straw and rice husk BC decreased Cd and As uptake and accumulation in alfalfa and cotton plants by causing As immobilization and decreasing its availability. El-Naggar et al. [153] claimed that BC transfers As into forms having good migration capability, while Wang et al. [120] also witnessed a reduction in As accumulation in maize with BC supplementation. In another study, BC (3%) application decreased the availability of both As and Cd, which enhanced its immobilization and conversion to less accessible forms [154]. The function groups present on BC surface promote the adsorption and complexation of As, leading to a reduction in As availability [155]. Arsenic interacts with functional groups present on the BC surface through electrostatic attraction [156]. Biochar also increases As desorption [157] and complexation with functional groups, which reduces As availability [158]. Some authors reported that Fe-modified BC increased As immobilization, which compensated the As desorption caused by the alkaline nature of BC [159,160]. Zang et al. [157] found that the co-precipitation peaks of As with Fe, Mn, and Ca show that precipitation is also an important mechanism used by BC to mitigate As availability. Similarly, Yao et al. [158] also found the precipitation of BC with Fe, Mn, Mg, and Ca, while Wang et al. [161] found no precipitation of BC with such minerals.

The anions such as PO_4_^3−^, SO_4_^2−^, and CO_3_^2−^ present in BC can exchange with anionic As. Since PO_4_ ions have a similar structure to As-V, they compete with As for absorption sites [162]. The presence of PO_4_ also promotes As desorption, thereby affecting As availability [163]. BC and iron-modified BC increased As fixation owing to an increase in Fe and different oxides of metals like Fe and Mn [146,164]. Biochar also decreases the specific and nonspecific binding ratio of As by converting the specific and nonspecifically bound As into amorphous and crystalline hydrated oxides [165]. Studies witnessed that higher Fe concentration in adoption systems leads to better fixation of As [166]. Biochar application increases the adsorption and fixation of As owing to the fact that BC increases Fe and Mn concentration. Both Fe and Mn improve the redox capacity of adsorbed materials, thus changing the As forms in soil and increasing its fixation and bioavailability [146].

#### 7.2.3. Biochar Improves Soil Microbial Activities and Biological Properties Under Arsenic Stress

Biochar improves soil enzymes and microbial activities to counter As toxicity. Studies have documented that phosphatase and urease enzymes are linked with hydrolytic enzyme systems. The activity of phosphatase is positively correlated with total P, while the activity of urease is positively linked with N [167]. Zhang et al. [133] noted that BC applied to As-polluted soil enhanced catalase, peroxidase, and urease activity by increasing SOM, nutrient availability, and strengthening the metabolic capacity of aerobic microorganisms [135]. The increase in catalase and urease activities with BC application is linked with reduced heavy metal availability [168].

Zhang et al. [146] found that BC showed a nonsignificant impact on microbial diversity in the first season, while, in the second season, it significantly impacted microbial diversity. Biochar improves soil bacterial diversity by decreasing availability of toxic metals in soil [169]. *Proteobacteria*, *Bacteroidetes*, and *Firmicutes* are considered to be core resistance phyla against heavy metals [170]. Biochar application is reported to increase the abundance of *Proteobacteria, Bacteroidetes*, and *Firmicutes* in As- and Cd-polluted soil [136], which is linked with reduced availability of both metals [171]. Different genera, such as *Proteobacteria* and *Sphingomonas*, can decrease As toxicity by oxidizing As-III into As-V [89]. BC increases the abundance of *Proteobacteria*, which mitigates the toxic impacts of As [47]. In another study, Sharifi-Rad et al. [92] noted a great increase in the abundance of *Proteobacteria* and *Bacteroidetes* and a decrease in *Actinobacteria* with BC in As-polluted soil. This increase in the abundance of the aforementioned bacteria was linked with improved SOM, nutrient availability, and a reduction in As availability [171]. The addition of BC affects soil properties and changes the microbial community structure; however, this effect could be varied over time and influenced by BC application.

Recently, Zhang et al. [135] found that BC and iron-modified BC reduced *Alicyclobacillaceae* abundance and increased the abundance of *Bacillus*. Another group of researchers found that BC and iron-oxide-modified BC altered soil microbial composition by changing the C:N ratio [172]. Tang et al. [173] reported that BC application enhanced the abundance of *Firmicutes* and *Proteobacteria* in mining areas [173]. Likewise, Qu et al. [174] documented that sulfur–iron-modified BC increased the abundance of *Actinobacteria*. Moreover, Yang et al. [175] found that iron-modified BC increased *Bacillus* abundance in As-polluted soil (141 mg kg^−1^), which, in turn, improved rice growth. Additionally, iron-modified BC shifted the abundance of *Arthrobacter* and *Gemmatimonas*, which promoted plant growth by regulating soil nutrients [176].

#### 7.2.4. Biochar Ensures Sustainable and Safe Crop Production in Arsenic-Polluted Soils

Biomass is an important factor in determining the toxic impacts of As on plants [177]. Arsenic toxicity decreases growth and biomass production by inducing oxidative damage and disturbing plant function. Biochar has shown significant results in improving plant growth and biomass in As-polluted soils. Hakeem et al. [107] found that BC increased root length by 24.48% and 19.76%, respectively, in 600 mg kg^−1^ As-polluted soil. In another study, cotton-shell-based BC (1 and 2%) significantly enhanced the growth and grain yield of quinoa by improving photosynthetic efficiency, antioxidant activities, and decreasing As availability [118]. Among them, 2% BC significantly enhanced plant growth and biomass production compared to 1%. Other researchers also reported an increase in growth and biomass in As-contaminated soils owing to improved nutrient and water uptake and As adsorption [178]. Biochar application restricted As-V in maize roots and its accumulation in upper plant parts, thereby leading to significant improvement in plant growth [120]. Biochar also improves root structure, which ensures better nutrient and water absorption that are conducive to better plant growth and development in As-stressed conditions [179].

Plant biomass is an important factor in determining the toxicity of As on plants [177]. Arsenic considerably decreased biomass production; however, BC application maintains a continuous supply of nutrients and protects the plants from oxidative damage, thereby increasing biomass production in stress conditions [180]. Biochar also improves soil enzymes and microbial activities, which helps in decreasing As toxicity and leads to better growth [109]. Biochar increases Fe plaque formation on the root surface and decreased As migration to the plant, thus ensuring better growth [181]. Biochar also reduced the mobility and availability of As in soil, leading to better growth and grain production [182].

Additionally, BC applied to As-polluted soils also increases biomass production by increasing soil pH, cation exchange capacity (CEC), and redox potential [183]. A biochar-mediated increase in soil pH decreases As availability by causing its fixation, thereby leading to better growth and biomass production [184]. Biochar also improves root growth and surface area, which ensures better nutrient absorption and leads to a substantial increase in crop productivity in As-contaminated soil [129]. Few studies assessed the effects of BC in mitigating the health risks of growing crops in As-polluted soils. For instance, Shabbir et al. [118] found that 2% BC effectively enhanced quinoa growth and yield and minimized the health risks of growing quinoa in As-polluted soil [118]. These authors also found that noncancer hazard quotient (HQ) and incremental lifetime cancer risk (ILTCR) values were higher than the threshold levels after using the quinoa grown with BC. The use of BC lowered the HQ values to a safer level (<1) and it also reduced the ILTCR value below the threshold level.

## 8. Integrative Application of Biochar and Other Amendments to Alleviate Arsenic Toxicity

Biochar is also used in combination with other amendments to remediate the As-polluted soils. Co-applying BC with other amendments has shown promising results in addressing As pollution. For instance, Liao et al. [60] applied BC along with *B. faecalis* to As-polluted soil and found a significant increase in plant growth, morphological traits, chlorophyll synthesis, and uptake and accumulation of N, P, and K under As stress (600 mg kg^−1^). A very recent study investigated the impacts of BC and silicon nanoparticles (NPs) on chili plants growing under As-polluted soil. They witnessed that co-applying BC Si-NPs substantially enhanced SOD and CAT activities by 55 and 66% and provided resistance against oxidative damages. Likewise, BC + Si-NPs also enhanced photosynthetic efficiency (52%), stomatal conductance (39%), and soluble sugars (42%) and reduced As accumulation in roots and straw by 61% and 37%, respectively. These findings also confirmed that co-applying BC+ Si-NPs protected the leaf ultra-structure and shielded the plants against As (7.5 mg kg^−1^, As-III) induced oxidative damage, thereby ensuring safe and better productivity [126]. Recently, Roy et al. [185] also studied the impacts of BC, vermin-compost (VC), and duckweed (DW) on rice plants growing in As-contaminated soil. Their findings indicated that combining BC, VC, and DW enhanced morphological growth, chlorophyll synthesis, grain yield (44.4%), and reduced oxidative stress. Further, a combination of these three amendments also reduced As accretion in roots, straw, and grains and bio-concentration and translocation factors below 1. These authors suggested that a combination of BC with VC and DW could be an important practice to ensure safer production and improve human health [175].

Shukla et al. [186] applied BC (10 t ha^−1^) and ZnO-NPs (80 mg L^−1^) to rice crop. They performed the foliar spray of ZnO-NPs at the heading and jointing stage and found that co-applying BC and ZnO-NPs enhanced chlorophyll synthesis and decreased EL (48–62%), MDA production (14–55%), and As accumulation in rice tissues and grains. They also reported that BC in combination with ZnO-NPs also enhanced APX, CAT, and SOD activities and protected the plants from oxidative damage. Likewise, Sattar et al. [121] also found that combining BC with Si reduced As accumulation in shoots (69%) and grains (142%) and improved rice productivity by increasing antioxidant activities and osmolyte synthesis. Mensah et al. [177] tested the impacts of BC, compost, iron oxide, manures, and inorganic fertilizers in improving the quality of higher contaminated As (1807 mg kg^−1^) contaminated soil. The manures, compost, and iron oxidative decreased the soil available P; likewise, iron oxide decreased the bioavailable As 93%, while compost, manure, inorganic fertilizer, and BC enhanced bioavailable As [187]. Recently, Kapoor et al. [134] studied the impacts of corncob BC and steel slag (SS) in mitigating As toxicity. Co-applying SS and BC improved okra growth and decreased oxidative damage and As accumulation in roots, which protected okra plants. The effects of BC and nitrate fertilizers were studied on As-contaminated soil. The authors reported that BC and nitrate fertilizers significantly reduced the As availability by 26.6% and facilitated the conversion of toxic As forms to less toxic forms. Co-applying BC and nitrate fertilizers also enhanced soil redox potential, organic matter, soil pH, and dissolved organic carbon. The same treatment combination also enhanced the abundance of *Bacillus*, *Arthrobacter*, and *Paenibacill* and these microbes favor the immobilization of As [188].

## 9. Practical Application, Challenges, and Perspectives of Biochar Application to Remediate Arsenic-Polluted Soils

The cost of raw materials and their reusability affect the practical use of BC. Generally, BC is produced from plant biomass and agricultural and industrial waste, and the origin of feedstock considerably affects BC properties. Biochar may contain toxic metals that affect human health and soil quality; therefore, these effects must be considered to ensure its optimal production [189]. Biochar contains a significant amount of toxic elements, dioxins, volatile organic compounds, and polycyclic aromatic hydrocarbons [190], which can pose serious threats to humans and other livings organisms [191]. Therefore, to produce a safe and cleaner BC production, great care must be taken while selecting the feedstock. The addition of contaminated BC can increase As mobility, posing serious health risks [191]. Biochar enriched in toxic metals also adversely affects plants due to the leaching of toxic metals. Visioli et al. [192] found that the higher electrical conductivity of BC and the presence of copper adversely impacted root growth and seed germination. Wu et al. [193] found that calcium-based magnetic BC increased the formation of Fe plaque owing to the presence of higher Fe from the modified BC. They found that 1% modified BC enhanced roots and shoots fresh weight and plant height, while 2% BC application decreased these traits. This indicates that the potential risks of using BC should be considered and phytotoxicity must also be noted before using the BC.

The application of BC alone may not be suitable for degraded and heavily disturbed soils lacking essential nutrients [132]. Beesley et al. [132] applied compost and BC to heavily contaminated soil and found that the combination of BC and compost decreased the As availability and enhanced nutrient availability and subsequent plant growth. Other authors found that combining BC and zero-valent Fe significantly remediated the moderate and highly As-contaminated soils [114]. Further, their combination also reduced As accumulation in seeds, possibly due to the conversion of effective As fractions into effective fractions. Generally, BC has a lower density as compared to water and it can float when applied under irrigation conditions, leading to loss of BC. Thus, BC can be used with other amendments to change its properties. Further, different types of BC-based materials, including millimeter-sized porous granular BC, BC-based bead, and BC-based hydrogel, are produced to counter this problem [88,194,195]. There is limited information on the use of BC as filler material; thus, studies are required to explore this area. Further, factors that affect the ability of BC for As adsorption are also very complex and interactive. The environmental conditions and modification significantly impact the adsorption of As. Nkoh et al. [196] studied the impacts of BC modification of the adsorption of heavy metals. They witnessed that different properties of BC, including, surface area, pH, pore volume, and ash contents, significantly affect BC performance, and these properties are also affected by different modifications.

## 10. Conclusions and Future Prospective

Arsenic significantly reduces plant growth and biomass production by disrupting plant functioning, proteins, and amino acid synthesis and increasing ROS. Biochar improves plant growth under As toxicity by improving plant morpho-physiological and biochemical functioning, antioxidant activities, and osmolyte production and maintaining nutrient homeostasis. Biochar also causes As immobilization and complexion and decreases its uptake and accumulation in plant tissues. This review presents insights to counteract As toxicity from soil and plant systems using biochar.

The impact of biochar on growth and biomass production is well documented, though its role in seed germination under As stress is poorly investigated. This calls to investigate the mechanisms mediated by biochar to improve seed germination. Arsenic toxicity negatively affects nutrient uptake; nevertheless, studies are needed to open its impacts on nutrient signaling and nutrient channels under As stress. It is also recommended that authors should study the anatomical changes mediated by biochar to counter As toxicity. Phyto-hormones play a crucial role in stress tolerance; nevertheless, the role of BC in hormonal balance in counteracting As toxicity is not explored yet. This calls to investigate this aspect in future studies to develop As-tolerant crops. The combination of BC with nanoparticles and microbes could be an important field to understand their interactions in reducing As toxicity. Further, genetic and molecular techniques like genetic engineering, QTL mapping, and omics must be investigated to understand the mechanisms mediated by biochar to counter As toxicity. The development of bio-sensors is also essential to assess the effectiveness of biochar in remediating As-polluted soils. Moreover, studies are also needed to determine the impacts of climate change, rainfall, and temperature fluctuations in mitigating As toxicity in soil–plant systems.

## Figures and Tables

**Table 2:** Effect of biochar application on growth, physiological, and biochemical activities and As uptake.

Plant Species	As Stress	Rate of BC	Effects on Plant	References
Maize	10 mg kg^−1^	5%	Biochar application decreased oxidative stress and MDA production by increasing SOD (46.55%), CAT (82.82%), and GST (153.83%) activity, flavonoid synthesis (75.37%), soluble sugars, amino acids, and nutrient availability and decreasing As uptake and accumulation.	[120]
Maize	600 mg kg^−1^	0.5%	Co-applying BC with bacteria enhanced plant height (99%), shoot (96%) and root dry biomass (91%), chlorophyll synthesis (94%), and N, P, and K concentration in plant tissues.	[108]
Maize	12 mg kg^−1^	50 g kg^−1^	Biochar increased plant height (2.91%), leaf area (24.41%), cob length (5.29%), grains/cob (9.73%), grain weight (11.24%), grain yield (9.91%), chlorophyll synthesis (11.35%), TSP (26.76%), FAA (26.50%), SS (46.95%), SOD (20.44%), POD (16.91%), CAT (12.78%), and APX (20%) activities and decreased MDA (39%) and H_2_O_2_ (28.05%) production and As concentration in shoots (31.03%) and grains (70.58%).	[121]
Quinoa	20 mg kg^−1^	1%	Biochar addition enhanced APX, CAT, and SOD activities, grain and biomass yield, chlorophyll synthesis, tissue, N, P, and K contents and reduced the As uptake, transport, and accumulation.	[122]
Quinoa	20 mg L^−1^	2%	Biochar increased the root and shoot lengths by 2.6%% and 2.4%, their dry weights by 2.9% and 0%, and grain yield by 30%. Further, BC also enhanced the RWC by 28%, stomatal conductance by 156%, chlorophyll contents by 2.8%, shoot and root K by 18% and 115%, and membrane stability by 136%. Additionally, BC also decreased As accretion in shoots (75%), roots (32%), and grains (95%) and increased SOD (33%), POD (31%), and CAT (34%) activities	[118]
Rice	60 mg kg^−1^	20 g kg^−1^	Biochar application decreased H_2_O_2_ production and enhanced the APX and CAT activities and N, P, K, and S concentration in plant tissues and decreased As accumulation.	[123]
Water Spinach	1 mg L^−1^	20 t ha^−1^	Biochar addition reduced As accumulation and improved plant growth and As adsorption.	[124]
Napier grass	68 mg kg^−1^	5%	BC application reduced As uptake and accumulation by causing stabilization and immobilization of As. Further, BC also improved the plant relative growth rates, biomass production, and chlorophyll synthesis.	[125]
Rice	100 µM	5%	Biochar application decreased ROS production and membrane damage and increased organic acids, proline synthesis, antioxidants activities, plant growth, biomass production, gas exchange characteristics, and decreased the As accumulation in plant tissues.	[112]
Rice	231 mg kg^−1^	3%	Biochar addition enhanced root, shoot, husk, and grain weight and decreased As accumulation in roots, husks, and grains. Biochar application also increased synthesis of glutamate, histidine, arginine, aspartate, serine, glycine, and proline and increased the abundance of Acidobacteria, Proteobacteria, Choloroflexi, Actinobacteria, and Firmicutes.	[126]
Rice	105 mg kg^−1^	5%	Biochar decreased As in roots, straw, and grain and increased As dilution and biomass production.	[127]
Rice	120 mg kg^−1^	3%	Biochar supply increased the As in soil solution and decreased As in amorphous Fe/Al oxide fraction. Biochar also increased the abundance of Fe-reducing bacteria, including Clostridum (27.3%), Bacillus (2.39%), and Caloramator (4.46%), and As-reducing (19%) genes.	[62]
Rice		1.6%	Biochar supplementation increased cation exchange capacity and reduced the As concentration in rice lower than 0.2 mg kg^−1^. Biochar supply also decreased As concentration in iron plaque, rice stems, leaves, husks, and roots.	[128]
Rice	138 mg kg^−1^	2%	Biochar application enhanced soil pH and CEC and reduced the bioavailable forms of As. Further, BC also converted the specifically bound forms of As into hydrous oxide bound and crystalline hydrous oxide forms and increased soil urease, catalase, phosphate, and peroxidase activities and abundance of Proteobacteria, Acidobacteria, and Gemmatimonadetes.	[129]
Rice	73 mg kg^−1^	2%	Biochar supply improved root growth and aboveground biomass and decreased the As accumulation in rice plant parts, which was linked with oxidation of As by Mn oxides. Biochar also enhanced amino acid synthesis and Mn concentration by 36%.	[130]
Chilli	7.5 mg kg^−1^	10 g kg^−1^	Biochar enhanced shoot (34.24%) and root length (50.47%) and their biomass (43.55 and 52.07%), chlorophyll synthesis, SOD (18.12%), CAT (15.78%), soluble sugars (37%), and protein (27.20%) and decreased soil As (52.42%) availability.	[131]
Tomato	3003 mg kg^−1^	30%	Biochar application reduced As accumulation in soil, water, roots, shoots, and fruits and increased water and soil pH, Fe availability, and plant fresh and dry biomass production.	[132]
Pak choi	1000 mg L^−1^	3%	Biochar application increases aboveground biomass, chlorophyll synthesis, RWC, APX, CAT, and POD activities and decreased MDA production and As accumulation in roots and stem.	[133]
Basil	100 mg kg^−1^	5%	Biochar enhanced soil organic matter, microbial biomass carbon, soil respiration, and soil enzyme activities (urease, alkaline phosphatase, and dehydrogenase) and decreased As availability.	[134]
Okra	10 mg kg^−1^	2 g kg^−1^	Biochar application decreased As accumulation in root and shoots of okra, increased antioxidant activities and performance of glyoxalase enzyme, and decreased methylglyoxal production. Further, BC also decreased oxidative damages and increased the synthesis of thiol and phytochelatins.	[135]

MDA: malondialdehyde, SOD: superoxide dismutase, CAT: catalase, GST: glutathione transferases, N: nitrogen, P: phosphorus, K: potassium, TSP: total soluble protein, FAA: free amino acids, SS: soluble sugars, APX: ascorbate peroxide, S: sulfur, ROS: reactive oxygen species, CEC: cation exchange capacity, RWC: relative water contents, BC: biochar, H_2_O_2_: hydrogen peroxide, S: sulfur, CEC: cation exchange capacity.
